# 新辅助免疫治疗联合化疗对IB-IIIB期非小细胞肺癌患者的疗效和安全性分析

**DOI:** 10.3779/j.issn.1009-3419.2025.106.16

**Published:** 2025-06-30

**Authors:** Zihao LI, Xin WANG, Yulong WANG, Zhuoer CUI, Xin WANG, Xiao LI, Guanchao JIANG, Xun WANG

**Affiliations:** ^1^100044 北京，北京大学人民医院胸外科，胸部肿瘤研究所，中国医学科学院早期非小细胞肺癌智能诊疗创新单元（李子豪，王鑫，王玉龙，崔卓尔，李晓，姜冠潮，王迅）; ^1^Department of Thoracic Surgery, China Thoracic Oncology Institute, Peking University People’s Hospital, Research Unit of Intelligence Diagnosis and Treatment in Early Non-small Cell Lung Cancer, Chinese Academy of Medical Sciences, Beijing 100044, China; ^2^300222 天津，天津市胸科医院胸外科（王新）; ^2^Department of Thoracic Surgery, Tianjin Chest Hospital, Tianjin 300222, China

**Keywords:** 肺肿瘤, 非小细胞肺癌, 新辅助治疗, 免疫治疗, Lung neoplasms, Non-small cell lung cancer, Neoadjuvant therapy, Immunotherapy

## Abstract

**背景与目的** 新辅助免疫治疗联合化疗已成为非小细胞肺癌（non-small cell lung cancer, NSCLC）的重要治疗手段。然而其实际应用经验尚不充分，许多临床因素与治疗获益的关系尚无定论。本研究旨在分析真实世界中新辅助免疫治疗联合化疗对IB-IIIB期NSCLC患者的有效性和安全性，评估不同临床特征下患者的生存情况，并识别病理学缓解的临床预测因素。**方法** 本研究纳入2019年8月至2024年3月在北京大学人民医院接受2-4个周期新辅助免疫治疗联合化疗后行肺癌根治术的IB-IIIB期NSCLC患者，通过收集病历资料及随访信息，分析其治疗反应、不良事件和生存情况。通过*Logistic*回归分析病理学缓解的临床预测因素。**结果** 共纳入183例患者，其中III期患者116例（63.4%）。39例（21.3%）患者出现3级及以上免疫相关不良事件（immune-related adverse events, irAEs）。118例（64.5%）患者达到影像学完全缓解（complete response, CR）或部分缓解（partial response, PR）。180例（98.4%）患者实现R0切除。107例（58.5%）患者达到主要病理缓解（major pathologic response, MPR），其中78例（42.6%）患者实现病理学完全缓解 （pathologic complete response, pCR）。鳞状细胞癌、影像学客观缓解（CR/PR）与病理缓解（pCR/MPR）有关。中位随访时间为22.1（四分位间距：18.3-32.2）个月，2年无事件生存（event-free survival, EFS）率和总生存（overall survival, OS）率分别为82.5%和90.4%。达到病理缓解（pCR/MPR）与生存期延长有关联。**结论** 在真实世界中，新辅助免疫治疗联合化疗对IB-IIIB期NSCLC患者安全有效；达到病理缓解（pCR/MPR）的患者从新辅助免疫治疗联合化疗中得到的生存获益更佳；鳞状细胞癌、影像学客观缓解（CR/PR）对病理缓解（pCR/MPR）存在预测作用。

## Abstract (en)

**Background and objective** Neoadjuvant immunochemotherapy has emerged as an indispensable therapeutic modality for non-small cell lung cancer (NSCLC). However, its clinical application experience remains limited, and the associations between various clinical factors and treatment benefits remain undefined. This study aims to evaluate the efficacy and safety of neoadjuvant immunochemotherapy in patients with stage IB-IIIB NSCLC in a real-world setting, analyze survival outcomes among subgroups with diverse clinical characteristics, and identify potential clinical predictive factors for pathological response.**Methods** This study included patients with stage IB-IIIB NSCLC who underwent radical lung resection after 2-4 cycles of neoadjuvant immunochemotherapy at Peking University People’s Hospital between August 2019 and March 2024. Medical records and follow-up information were collected to analyze therapeutic response, adverse events and survival outcomes. *Logistic* analysis was used to identify clinical predictors of pathological response.**Results** Among 183 enrolled patients, 116 (63.4%) were stage III. Grade 3-4 immune-related adverse events (irAEs) occurred in 39 (21.3%) patients. Radiographic complete response (CR) or partial response (PR) was achieved in 118 (64.5%) patients. R0 resection was achieved in 180 (98.4%) patients. Major pathologic response (MPR) was observed in 107 (58.5%) patients, with 78 (42.6%) achieving pathologic complete response (pCR). Squamous cell carcinoma and radiographic objective response were associated with pathological response (pCR/MPR). With a median follow-up of 22.1 [interquartile range (IQR): 18.3-32.2] months, the 2-year event-free survival (EFS) and overall survival (OS) rates were 82.5% and 90.4%, respectively. Achievement of pathological response (pCR/MPR) was correlated with prolonged survival outcomes.**Conclusion** Neoadjuvant immunochemotherapy is safe and effective for patients with stage IB-IIIB NSCLC. Patients achieving pCR or MPR exhibit significantly better survival benefits from neoadjuvant immunochemotherapy. Squamous cell carcinoma and radiographic objective response can serve as clinical predictors of pathological response.

肺癌的发病率和死亡率在我国各类恶性肿瘤中均居首位^[1]^。其中，非小细胞肺癌（non-small cell lung cancer, NSCLC）约占总病例数的85%^[2]^。根据美国国立综合癌症网络（National Comprehensive Cancer Network, NCCN）指南，手术仍是IA-IIIA期NSCLC最有效的治疗方法^[3]^。但在过去，接受根治手术后患者的总体生存状况仍不理想^[4]^。新辅助治疗对于可切除和潜在可切除的NSCLC患者，能够起到缩瘤、降期和消灭微转移灶的作用，从而改善患者的长期生存。然而，传统新辅助化疗仅能将患者的5年生存率提升约5%^[5]^。

随着肿瘤免疫治疗研究的深入，程序性死亡受体1（programmed death 1, PD-1）和程序性死亡配体1（programmed death ligand 1, PD-L1）抑制剂为NSCLC的综合治疗开辟了新的天地^[6]^。临床试验^[7,8]^结果表明，对于早期和局部进展期NSCLC，相比单纯新辅助化疗，新辅助免疫治疗联合化疗不仅能显著改善临床疗效，还具有良好的可耐受性。然而在真实世界中，新辅助免疫治疗联合化疗的应用经验尚不充分，对于具有不同临床特征的患者存在疗效差异。病理类型、肿瘤分期、治疗周期数等诸多临床因素与治疗获益的关系均存在较大争议^[9-12]^。这些问题亟待进一步探索，从而更好地指导临床实践。

本研究旨在通过分析真实世界数据，评估新辅助免疫治疗联合化疗对IB-IIIB期NSCLC患者的有效性和安全性，对比不同临床特征下患者的生存情况，并对病理学缓解的临床预测因素进行初步探索。

## 1 资料与方法

### 1.1 研究对象

本研究回顾性收集了2019年8月至2024年3月在北京大学人民医院胸外科行新辅助免疫治疗联合化疗的肺癌病例。患者均经至少由胸外科、肿瘤内科及影像科专家组成的多学科团队评估以确认治疗方案的可行性。纳入标准：（1）经病理学确诊的原发性NSCLC；（2）初治时临床分期为IB-IIIB期；（3）接受2-4个周期新辅助免疫治疗联合化疗；（4）新辅助治疗后接受了根治性肺癌切除术联合系统性淋巴结清扫。排除标准：（1）既往有肺癌病史；（2）已知合并其他恶性肿瘤；（3）术前接受其他抗肿瘤治疗；（4）术后病理证实为神经内分泌肿瘤；（5）临床资料严重缺失；（6）失访。

### 1.2 新辅助治疗

新辅助治疗方案均采用一种PD-1/PD-L1抑制剂联合含铂双药化疗，每3周为1个周期。每2个治疗周期后复查胸部计算机断层扫描（computed tomography, CT）。新辅助治疗结束后、接受根治手术前均需再次接受胸部增强CT或正电子发射计算机断层扫描（positron emission tomography/CT, PET/CT）检查。多学科团队通过评估药物疗效、不良反应及病灶可切除性来动态调整治疗方案，并确定最佳手术时机。

### 1.3 手术治疗

所有入组患者均接受了根治性肺癌切除术联合系统性淋巴结清扫。复杂手术定义为复合肺叶切除术、袖式肺叶切除术及全肺切除术。手术入路及切除范围由两位胸外科资深专家基于术前影像共同决策。术中送支气管残端及血管切缘进行冰冻快速病理检查，以确认是否达到R0切除。

### 1.4 疗效评价

依照实体瘤疗效评价标准（Response Evaluation Criteria in Solid Tumors, RECIST）1.1版，影像资料均由2名高年资放射科医师独立判读，存在争议时则向第3位资深放射科专家寻求参考。病理诊断分别由2名高年资病理科医师独立进行，存在争议时则向第3位资深病理科专家寻求参考。手术标本包括原发肿瘤及清扫的淋巴结。新辅助治疗反应通过计算存活肿瘤细胞的平均占比进行量化分析，病理学完全缓解（pathologic complete response, pCR）定义为原发病灶及淋巴结内均无残存肿瘤细胞，主要病理缓解（major pathologic response, MPR）定义为原发灶中残存肿瘤细胞比例≤10%。肿瘤分期参照美国癌症联合委员会（American Joint Committee on Cancer, AJCC）第8版标准，分别于新辅助治疗前、新辅助治疗后及手术后进行3次独立评估。

### 1.5 数据搜集与随访

患者资料主要通过调阅病历和电话访问来收集，主要内容包括人口学特征、病史、诊疗情况及随访信息等。术后首年内，随访分别于第1、3、6、9、12个月进行，术后第2年起调整为半年期随访，术后第3年起改为年度随访。首次随访的主要内容为患者围术期状况和后续治疗计划，此后的随访重点为患者的生存和复发情况。根据《常见不良事件评价标准5.0》评估新辅助治疗的安全性。根据Clavien-Dindo分级标准评估围术期并发症的严重性。局部复发定义为在原发肿瘤的同侧胸腔内（包括同侧肺门/纵隔淋巴结）复发。无事件生存期（event-free survival, EFS）的计算起点为病理确诊时间，终点事件包括：因疾病进展导致无法手术、复发或全因死亡，以先达到者为准。随访截止时间为2025年3月30日。本研究遵循《赫尔辛基宣言》（2013年修订版）进行，并通过北京大学人民医院伦理委员会批准（批准号：PHB271-001）。本研究为非干预性研究，因此知情同意被豁免。

### 1.6 统计学分析

采用SPSS 26.0统计软件进行数据整理和分析。计量资料若符合正态分布，采用均数 +标准差表示，两组的组间比较采用两独立样本t检验；若数据分布不符合正态分布，采用中位数和四分位数间距（interquartile range, IQR）（Q_L_, Q_U_）表示，两组的组间比较采用Mann-Whitney U检验。计数资料采用频数（百分比）表示，组间比较采用卡方检验或Fisher精确检验。采用*Kaplan-Meier*法的Log-rank（Mantel-Cox）检验进行生存分析，并绘制生存曲线图。采用*Logistic*回归分析进行单因素和多因素分析，单因素分析时*P*<0.05的变量纳入多因素分析。*P*<0.05为差异有统计学意义。

## 2 结果

### 2.1 一般资料

共收集了244例于2019年8月至2024年3月在北京大学人民医院胸外科行新辅助免疫治疗联合化疗的NSCLC患者资料，所有患者新辅助治疗后均接受了手术治疗，初筛后共189例符合纳入标准，其中6例因高龄、通气功能重度损减仅接受肺楔形切除术而被排除，最终共入组183例新辅助免疫治疗联合化疗后行根治性肺癌切除术的患者（图1）。

**图1:**
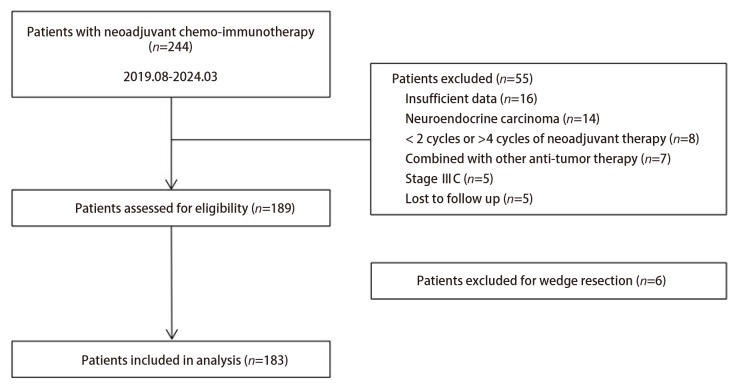
研究筛选流程图

患者基线资料见表1。根据病理类型将患者分为鳞癌组和腺癌组。本研究中患者以男性（161例，88.0%）、有吸烟史（100例，54.6%）者为主，原发肺癌以周围型（110例，60.1%）为主，初治时肿瘤分期以III期为主（116例，63.4%），其中IIIA期77例（42.1%），IIIB期39例（21.3%）。110例周围型肺癌中I期9例（腺癌2例，鳞癌7例）；II期28例（腺癌13例，鳞癌15例）；III期73例（腺癌32例，鳞癌41例）。73例中央型肺癌中I期15例（腺癌4例，鳞癌11例），II期15例（腺癌5例，鳞癌10例），III期43例（腺癌7例，鳞癌36例）。组间比较显示，鳞癌组中有吸烟史的患者比例显著高于腺癌组（60.8% *vs* 42.9%, *P*=0.020）；腺癌组中女性（28.6% *vs* 3.3%, *P*<0.001）、周围型肺癌（74.6% *vs* 52.5%, *P*=0.004）、PD-L1肿瘤阳性比例分数（tumor proportion score, TPS）<1%（27.0% *vs* 8.3%, *P*<0.001）的患者比例显著高于鳞癌组。18例（9.8%）患者术后基因检测结果显示表皮生长因子受体（epidermal growth factor receptor, *EGFR*）或间变性淋巴瘤激酶（anaplastic lymphoma kinase, *ALK*）基因突变，腺癌组已知突变比例显著高于鳞癌组（23.8% *vs* 2.5%, *P*<0.001）。除此之外，两组患者的基线特征并无显著差异。免疫检查点抑制剂和化疗药物的使用情况见表2。

**表1:** 接受新辅助免疫治疗联合化疗的NSCLC患者的基线特征

Variables	All (*n*=183)	ADC (*n*=63)	SCC (*n*=120)	P
Gender, *n* (%)				<0.001
Male	161 (88.0)	45 (71.4)	116 (96.7)	
Female	22 (12.0)	18 (28.6)	4 (3.3)	
Age (yr), median (IQR)	63 (58, 69)	63 (58, 68)	63 (58, 69)	0.530
BMI (kg/m^2^)	24.4±3.0	24.7±3.0	24.2±3.1	0.276
Smoking status, *n* (%)				0.020
Never	83 (45.4)	36 (57.1)	47 (39.2)	
Current or ever	100 (54.6)	27 (42.9)	73 (60.8)	
Major comorbidity, *n* (%)				
Bronchial asthma	1 (0.5)	1 (1.6)	0 (0.0)	0.344
COPD	13 (7.1)	6 (9.5)	7 (5.8)	0.535
Hypertension	75 (41.0)	25 (39.7)	50 (41.7)	0.795
CAD	34 (18.6)	12 (19.0)	22 (18.3)	0.906
Diabetes	25 (13.7)	7 (11.1)	18 (15.0)	0.467
Tumor location, *n* (%)				0.004
Central	73 (39.9)	16 (25.4)	57 (47.5)	
Peripheral	110 (60.1)	47 (74.6)	63 (52.5)	
Tumor stage, *n* (%)				0.362
Stage I	24 (13.1)	6 (9.5)	18 (15.0)	
Stage II	43 (23.5)	18 (28.6)	25 (20.8)	
Stage III	116 (63.4)	39 (61.9)	77 (64.2)	
Tumor size, *n* (%)				0.284
<4 cm	83 (45.4)	32 (50.8)	51 (42.5)	
≥4 cm	100 (54.6)	31 (49.2)	69 (57.5)	
PD-L1 TPS (%)				<0.001
<1%	27 (14.8)	17 (27.0)	10 (8.3)	
1%-49%	35 (19.1)	17 (27.0)	18 (15.0)	
≥50%	16 (8.7)	4 (6.3)	12 (10.0)	
Unknown	105 (57.4)	25 (39.7)	80 (66.7)	
*EGFR* or *ALK* mutations, *n* (%)				<0.001
Positive	18 (9.8)	15 (23.8)	3 (2.5)	
Negative or unknown	165 (90.2)	48 (76.2)	117 (97.5)	
Treatment cycles, *n* (%)				0.259
2	26 (14.2)	9 (14.3)	17 (14.2)	
3	109 (59.6)	42 (66.7)	67 (55.8)	
4	48 (26.2)	12 (19.0)	36 (30.0)	

NSCLC: non-small cell lung cancer; ADC: adenocarcinoma; SCC: squamous cell carcinoma; IQR: interquartile range; BMI: body mass index; COPD: chronic obstructive pulmonary disease; CAD: coronary artery disease; PD-L1: programmed death ligand 1; TPS: tumor proportion score; *EGFR*: epidermal growth factor receptor; *ALK*: anaplastic lymphoma kinase.

**表2:** 不同免疫检查点抑制剂和化疗方案的使用情况

Chemotherapy	Immunotherapy						
	Pembrolizumab	Tislelizumab	Toripalimab	Sintilimab	Nivolumab	Durvalumab	Camrelizumab
Paclitaxel+Carboplatin	55	37	31	5	4	2	1
Pemetrexed+Carboplatin	15	15	13	1	2	0	0
Paclitaxel+Cisplatin	0	0	1	0	0	0	0
Pemetrexed+Cisplatin	1	0	0	0	0	0	0

The numerical data in the table indicate the number of patients treated with corresponding drugs.

### 2.2 疗效与安全性

所有患者在新辅助治疗结束后、接受根治手术前均完成了基于RECIST 1.1标准的影像学再评估，结果（表3）显示：完全缓解（complete response, CR）10例（5.5%），部分缓解（partial response, PR）108例（59.0%），病情稳定（stable disease, SD）61例（33.3%），病情进展（progressive disease, PD）4例（2.2%）。客观缓解率（objective response rate, ORR）为64.5%。术后病理显示：107例（58.5%）患者达到MPR，其中78例（42.6%）实现pCR。鳞癌组MPR率（66.7% *vs* 42.9%, *P*=0.002）和pCR率（49.2% *vs* 30.2%, *P*=0.013）均显著高于腺癌组。腺癌组N2期患者术后N分期保持不变的比例显著高于鳞癌组（23.8% *vs* 5.8%, *P*=0.002）。此外，18例已知*EGFR*或*ALK*基因突变的患者中仅4例达到pCR或MPR，其病理缓解率显著低于*EGFR*或*ALK*突变阴性或未知者（22.2% *vs* 62.4%, *P*=0.001）。图2展示了患者总体的肿瘤原发灶-淋巴结-转移（tumor-node-matastasis, TNM）分期和N分期降期情况。

**表3:** 新辅助免疫治疗联合化疗的影像学和病理学疗效

Variables	All (*n*=183)	ADC (*n*=63)	SCC (*n*=120)	P
Clinical response, *n* (%)				0.068
CR	10 (5.5)	1 (1.6)	9 (7.5)	
PR	108 (59.0)	34 (54.0)	74 (61.7)	
SD	61 (33.3)	25 (39.6)	36 (30.0)	
PD	4 (2.2)	3 (4.8)	1 (0.8)	
MPR, *n* (%)				0.002
No	76 (41.5)	36 (57.1)	40 (33.3)	
Yes	107 (58.5)	27 (42.9)	80 (66.7)	
pCR, *n* (%)				0.013
No	105 (57.4)	44 (69.8)	61 (50.8)	
Yes	78 (42.6)	19 (30.2)	59 (49.2)	
Change in pN stage, *n* (%)				0.002
N0→N0	46 (25.1)	12 (19.0)	34 (28.3)	
N1→N1	12 (6.6)	5 (7.9)	7 (5.8)	
N1→N0	19 (10.4)	3 (4.8)	16 (13.3)	
N2→N2	22 (12.0)	15 (23.8)	7 (5.8)	
N2→N1	8 (4.4)	4 (6.3)	4 (3.3)	
N2→N0	65 (35.5)	18 (28.6)	47 (39.3)	
N3→N2	1 (0.5)	1 (1.6)	0 (0.0)	
N3→N0	2 (1.1)	0 (0.0)	2 (1.7)	
Progress	8 (4.4)	5 (8.0)	3 (2.5)	

CR: complete response; PR: partial response; SD: stable disease; PD: progressive disease; MPR: major pathologic response; pCR: pathological complete response.

**图2:**
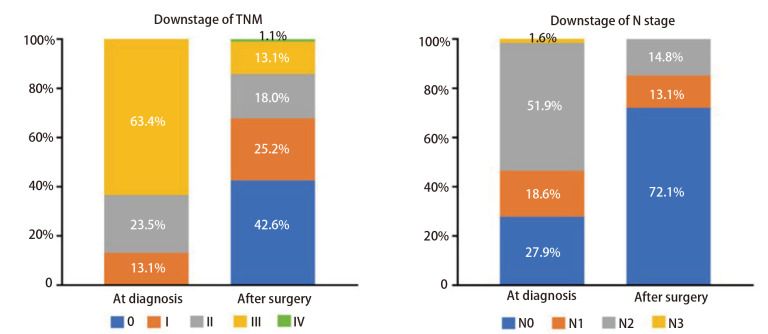
TNM分期及N分期降期情况

新辅助治疗全程未见致死性不良事件，共在39例（21.3%）患者中观察到42例次3级及以上免疫治疗相关不良事件（immune-ralated adverse events, irAEs），最常见的AEs为骨髓抑制，共29例（15.8%）；其他AEs包括：皮肤病变和药物性肝损伤各4例（2.2%），免疫相关性肺炎2例（1.1%），免疫性心肌炎、甲状腺功能减退、肾上腺皮质功能减退各1例（0.5%）。

### 2.3 手术结果

中位手术间隔时间为37（IQR: 30, 49）d，中位手术时长为160（IQR: 135, 205）min，中位术中出血量为50（IQR: 20, 100）mL，中位胸引管留置时长为4（IQR: 3, 5）d，中位住院时长为12（IQR: 10, 15）d。共172例（94.0%）患者顺利实施电视辅助胸腔镜手术（video-assited thoracoscopic surgery, VATS），7例（3.8%）患者因胸腔镜下难以完成手术而中转开胸，4例（2.2%）患者直接进行了开胸手术。40例（21.9%）患者接受了复杂手术。180例（98.4%）患者实现了R0切除。上述指标在鳞癌组与腺癌组间未见统计学差异（表4）。

**表4:** 新辅助免疫治疗联合化疗后的手术结果汇总

Variables	All (*n*=183)	ADC (*n*=63)	SCC (*n*=120)	P
Interval to surgery (d), median (IQR)	37 (30, 49)	36 (29, 48)	37 (30, 50)	0.429
Surgical approach, *n* (%)				0.999
VATS	172 (94.0)	60 (95.2)	112 (93.3)	
Thoracotomy	4 (2.2)	1 (1.6)	3 (2.5)	
VATS conversion to thoracotomy	7 (3.8)	2 (3.2)	5 (4.2)	
Extent of surgery, *n* (%)				0.069
Lobectomy	143 (78.1)	54 (85.7)	89 (74.2)	
Bilobectomy	13 (7.1)	4 (6.3)	9 (7.5)	
Pneumonectomy	11 (6.1)	4 (6.3)	7 (5.8)	
Sleeve lobectomy	16 (8.7)	1 (1.7)	15 (12.5)	
R0 resection, *n* (%)	180 (98.4)	60 (95.2)	120 (100.0)	0.302
Operation duration (min), median (IQR)	160 (135, 205)	150 (130, 185)	170 (140, 210)	0.209
Blood loss (mL), median (IQR)	50 (20, 100)	50 (20, 100)	50 (20, 100)	0.574
Chest tube duration (d), median (IQR)	4 (3, 5)	3 (3, 5)	4 (3, 5)	0.278
LOS (d), median (IQR)	12 (10, 15)	12 (10, 15)	12 (10, 15)	0.857

NAT: neoadjuvant therapy; VATS: video-assisted thoracoscopic surgery; LOS: length of stay.

30 d内再入院率为1.5%（3例，不包括因接受辅助治疗入院），原因分别为呼吸衰竭（1例，0.5%）、严重肺部感染（2例，1.0%）。术后30 d内死亡率为0.5%（1例），死因为呼吸衰竭。II-IV级围术期并发症共22例（12.0%），最常见的并发症为术中出血量≥400 mL，共10例（5.5%），其他并发症包括：肺部感染4例（2.2%）；乳糜胸、大量胸腔积液、肺不张各2例（1.1%）；急性肝损伤、纵隔气肿各1例（0.5%）。

### 2.4 生存分析

中位随访时间为22.1（IQR: 18.3, 32.2）个月，中位EFS和总生存期（overall survival, OS）未达到。总体的2年EFS率和OS率分别为82.5%（95%CI: 76.6%-88.8%）和90.4%（95%CI: 85.6%-95.3%）。全人群的*Kaplan-Meier*生存曲线见图3。随访期间，共34例（18.5%）患者出现肿瘤复发，其中16例（8.7%）为局部复发，18例（9.8%）为远处转移。共18例（9.8%）患者死亡，其中12例（6.6%）死因为肿瘤进展，2例（1.1%）死因为术后并发症，1例（0.5%）死因为辅助治疗并发症，3例（1.6%）死于与原发肿瘤及治疗无关的因素。

**图3:**
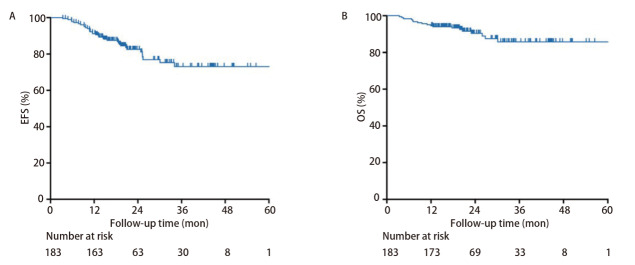
全人群*Kaplan-Meier*生存曲线。 A：EFS；B：OS。

亚组分析（图4）显示，MPR组的EFS和OS均显著优于非MPR组，风险比（hazard ratio, HR）分别为0.20（95%CI: 0.09-0.42, *P*<0.001）和0.18（95%CI: 0.06-0.54, *P*=0.002）；pCR组的EFS显著优于非pCR组，HR为0.11（95%CI: 0.03-0.36, *P*<0.001）；pCR组的OS较非pCR组有获益趋势，但两组的生存曲线并无显著差异，HR为0.37（95%CI: 0.12-1.11, *P*=0.076）。鳞癌组和腺癌组的EFS与OS均无统计学差异（图5），HR分别为0.64（95%CI: 0.33-1.26, *P*=0.198）和1.35（95%CI: 0.48-3.78, *P*=0.571）；早期组和局部进展期组的EFS和OS均无统计学差异，HR分别为1.00（95%CI: 0.50-2.02, *P*>0.999）和0.85（95%CI: 0.33-2.20, *P*=0.741）。

**图4:**
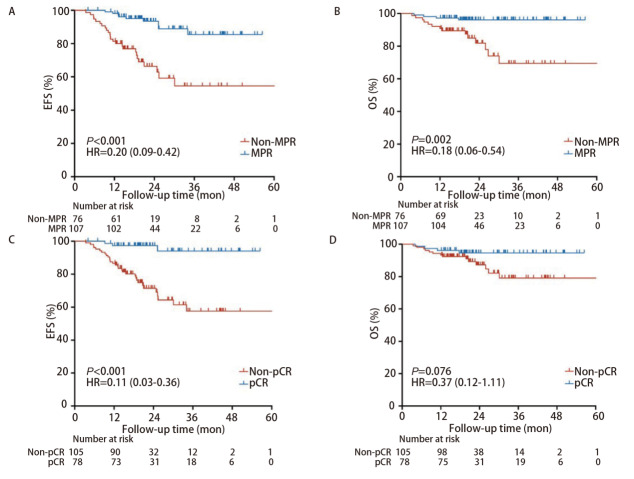
根据病理反应分组绘制的*Kaplan-Meier*生存曲线。 A：MPR组与非MPR组的EFS；B：MPR组与非MPR组的OS；C：pCR组与非pCR组的EFS；D：pCR组与非pCR组的OS。

**图5:**
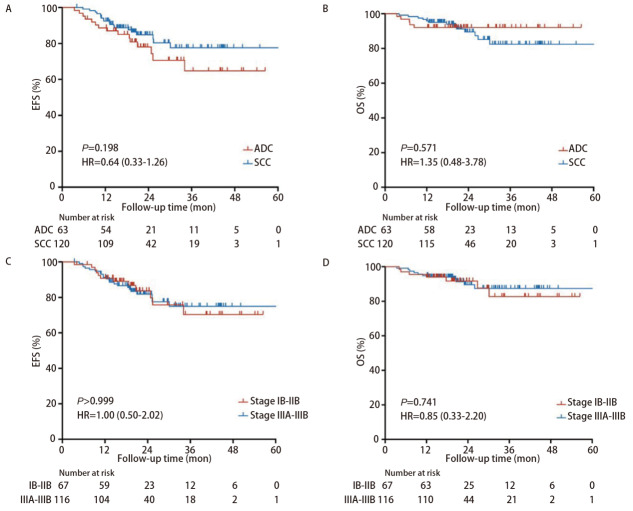
根据病理类型和基线临床分期分组绘制的*Kaplan-Meier*生存曲线。 A：ADC组与SCC组的EFS；B：ADC组与SCC组的OS；C：IB-IIB期组与IIIA-IIIB期组的EFS；D：IB-IIB期组与IIIA-IIIB期组的OS。

### 2.5 病理缓解的临床预测因素

为了识别病理缓解（pCR/MPR）的潜在预测指标将多个临床变量纳入单因素分析（表5），结果发现病理类型为鳞癌、影像学客观缓解可能与MPR和pCR有关（*P*<0.05）；将其进一步纳入多因素分析（表5），结果发现上述两项临床指标与MPR和pCR有明显关联（*P*<0.05）。

**表5:** MPR和pCR临床预测因素的*Logistic*回归分析

Variables		MPR								pCR						
		Univariate analysis				Multivariate analysis				Univariate analysis				Multivariate analysis		
		OR	95%CI	P		OR	95%CI	P		OR	95%CI	P		OR	95%CI	P
Age	<65 yr* *vs* ≥65 yr	1.562	0.861-2.835	0.142						1.333	0.740-2.402	0.338				
Gender	Male* *vs* Female	0.445	0.180-1.102	0.080						0.355	0.125-1.007	0.052				
Smoking history	No* *vs* Yes	1.149	0.637-2.073	0.645						1.132	0.628-2.041	0.679				
Tumor size	<4 cm* *vs* ≥4 cm	0.875	0.484-1.581	0.658						0.602	0.333-1.087	0.092				
Tumor location	C* *vs* P	0.883	0.484-1.613	0.687						1.011	0.556-1.839	0.972				
Histology	ADC* *vs* SCC	2.667	1.425-4.992	0.002		2.487	1.316-4.702	0.005		2.240	1.174-4.274	0.014		2.080	1.079-4.009	0.029
cN stage	<N2* *vs* ≥N2	0.676	0.373-1.225	0.197						0.651	0.361-1.174	0.154				
PD-LI TPS	<1%* *vs* ≥1%	1.787	0.722-4.423	0.210						2.182	0.837-5.686	0.110				
NAT cycles	<3* *vs* ≥3	1.246	0.541-2.868	0.606						1.015	0.438-2.351	0.972				
Objective response	No* *vs* Yes	2.191	1.182-4.063	0.013		2.003	1.063-3.774	0.032		2.175	1.149-4.119	0.017		2.016	1.053-3.858	0.034

*indicates baseline groups of variables. OR: odds ratio; CI: confidence interval; C: central; P: peripheral.

## 3 讨论

免疫检查点抑制剂通过其可控的毒副作用和持久的抗肿瘤效应，显著改善了NSCLC患者的长期预后^[13]^，免疫治疗已经成为NSCLC综合治疗体系中的重要环节^[14]^。本研究对北京大学人民医院胸外科接受新辅助免疫治疗联合化疗后行根治性肺癌切除术的NSCLC患者的基本特征、临床疗效、手术指标、不良反应、复发和生存情况进行了全面分析，并探索了病理学缓解的预测因素。

与既往临床试验^[9-11]^相比，本研究纳入了部分IB期NSCLC患者（24例，13.1%），尽管NCCN指南^[3]^并不建议对IB期患者进行新辅助治疗，但在临床实践中，部分IB期、中央型肺癌患者肿瘤邻近叶支气管开口，将为手术操作带来困难，并增加手术潜在风险。对于此类患者，为更好地规避这一问题，经本中心资深临床医生评估，并结合患者意愿，选择先进行新辅助免疫联合化疗。经统计，上述患者均顺利完成胸腔镜手术，并实现R0切除。

研究数据^[15-17]^显示，新辅助免疫治疗联合化疗后pCR率可达18.1%-62.9%；MPR率可达36.9%-79.5%。本研究中pCR率和MPR率分别为42.6%和58.5%，其中，鳞癌组的pCR率（49.2% *vs* 30.2%, *P*=0.013）和MPR率（66.7% *vs* 42.9%, *P*=0.002）均显著高于腺癌组，这可能与以下因素有关：（1）肺鳞癌中有吸烟史的患者比例更高，吸烟导致的DNA损伤会增加肿瘤突变负荷（tumor mutational burden, TMB），产生更多新抗原，从而增强免疫原性^[18]^；（2）肺鳞癌患者中约75%为高免疫亚型，其总T细胞、CD8^+ ^T细胞、自然杀伤（natural killer, NK）细胞、表达干扰素-γ（interferon-γ, IFNγ）的T细胞浸润程度均更高，直接增强了免疫系统的抗肿瘤能力^[19]^；（3）我国肺腺癌患者*EGFR*突变率更高，从而影响免疫治疗效果^[20]^。本研究通过*Logistic*回归分析发现鳞癌是病理缓解（pCR/MPR）的独立预测因素，这为新辅助免疫联合化疗的患者选择问题提供了重要参考。然而，目前对于此问题尚无明确结论，不同研究中并未显示出一致结果^[12,14]^，相信未来研究能做出更深入的解释。

新辅助免疫治疗联合化疗后的ORR通常为58%-78%^[21,22]^，本研究中ORR为64.5%。术后病理显示，10例影像学CR的患者均实现了pCR；108例影像学PR的患者中，有67例（62.0%）实现了pCR/MPR；同时发现影像学客观缓解（CR+PR）与病理缓解（pCR/MPR）存在显著关联，既往同样有研究^[23]^提示了二者间的潜在关联。然而，由于炎症、水肿和免疫细胞浸润等因素，新辅助免疫治疗可能导致肿瘤“假性进展”^[24]^，本研究亦存在相似现象，4例影像学PD患者中，1例术后病理证实达到了pCR。此外，CT技术的局限性也会对结果造成影响，如难以测量片状或条索状病灶、难以区分活性肿瘤与治疗后纤维化或坏死、难以发现微小残留病灶等。综上所述，影像学缓解情况对病理疗效具有预测作用，但其准确性受到一定限制，对于影像结果应谨慎解读。

虽然目前普遍认为3-4个周期的新辅助免疫治疗联合化疗可以最大化临床获益并灵活把握手术时机^[25]^，但关于治疗周期数与病理缓解率的关系尚无明确结论^[26]^。NeoSCORE首次探索了这一问题，研究显示，3周期方案的MPR率在数值上高于2周期方案，但结果无统计学差异^[27]^，一项真实世界临床研究也获得了相似的结果^[28]^。而一项meta分析^[29]^表明，治疗周期数对病理缓解率并无显著影响，本研究亦未发现两者间的显著关联。在临床实践中部分病例接受2-3个周期新辅助治疗后复查CT即发现肿瘤明显退缩，患者可能会选择不进行后续新辅助治疗而直接手术。因此，相比于寻求普适的最佳周期数，根据患者情况制定个体化方案，可能是此问题的更优解。

既往III期临床试验^[9-11]^显示，不同PD-L1表达水平下均可观察到病理缓解获益，且二者存在正相关趋势。然而在本研究中，对比PD-L1阴性并未发现PD-L1阳性为病理缓解的独立促进因素。在真实世界中考虑到经济条件等因素，许多患者并不愿意接受PD-L1检测，较多的缺失值（57.4%的患者PD-L1表达情况未知）可能是本研究与临床试验结果不一致的原因。

存在*EGFR*或*ALK*基因突变的肿瘤微环境理论上将对免疫治疗起负面作用^[30]^，目前并不建议将免疫检查点抑制剂用于驱动基因突变患者的常规治疗^[31]^，在LCMC3试验中*EGFR*突变患者均未达到MPR^[32]^，本研究中亦有类似情况，18例已知*EGFR*或*ALK*基因突变患者的病理缓解率显著低于突变阴性或未知者（22.2% *vs* 62.4%, *P*=0.001），然而，此结果基于小样本得出，新辅助免疫治疗对驱动基因突变患者的临床价值尚需进一步研究验证。

既往文献^[14,33]^显示，新辅助免疫治疗期间≥3级irAEs的发生率为8.0%-33.5%。本研究仅在21.3%的患者中观察到≥3级irAEs，且未见治疗相关死亡。为保证新辅助免疫治疗的最大获益，同时给患者足够的恢复时间，手术间隔时间通常为28-42 d^[12]^。本研究的中位手术间隔时间为37 d，手术率为100.0%，R0切除率高达98.4%，II-IV级围术期并发症发生率仅12.0%，术后30 d内死亡率仅0.5%，30 d内再入院率仅1.5%，上述水平与国内外研究^[34,35]^基本一致。这种安全优势为临床实践中风险效益比的评估提供了重要参考。

尽管CheckMate-816^[14]^首次证实了新辅助免疫联合化疗的EFS获益，此治疗模式的长期生存结果仍需更多真实世界数据验证。本研究的2年EFS率和OS率分别达到了82.5%和90.4%，进一步体现出新辅助免疫治疗联合化疗所带来的生存优势。一项meta分析^[36]^表明，新辅助治疗后达到病理缓解（pCR/MPR）与EFS和OS获益有关，本研究中MPR患者的EFS和OS均显著优于未达到MPR者，pCR患者的EFS显著优于非pCR者，再次显示出病理缓解（pCR/MPR）作为NSCLC患者生存替代终点的可行性。本研究中并未观察到pCR与非pCR患者OS的显著差异，这可能与以下因素有关：（1）非pCR患者中包含了MPR患者，使得整体生存情况有所改善；（2）随访时间相对较短，在许多非pCR患者中尚不足以观察到死亡事件。部分III期临床试验^[11,12]^对比了不同病理类型和临床分期患者的生存获益情况，并未显示出一致的结果。本研究中亦未见到鳞癌和腺癌、早期和局部进展期患者间的显著生存差异。结合目前证据，病理类型和临床分期可能并非影响患者长期生存的关键因素，同时也表明，局部进展期NSCLC患者可以从新辅助免疫治疗联合化疗中获得与早期患者相似的生存获益。

本研究存在一定的局限性，如仅基于单中心资料，难免存在患者选择偏倚；随访时间相对较短，长期生存尚需进一步验证；PD-L1表达情况和驱动基因状态缺失较多，可能对结果造成一定影响。未来本课题组将整合多中心数据开展研究，并持续更新生存情况。

综上所述，在真实世界中新辅助免疫治疗联合化疗对IB-IIIB期NSCLC患者安全有效；达到病理缓解（pCR/MPR）的患者从新辅助免疫治疗联合化疗中得到的生存获益更佳；鳞状细胞癌、影像学客观缓解（CR/PR）对病理缓解（pCR/MPR）存在预测作用。
